# The DNA damage checkpoint: A tale from budding yeast

**DOI:** 10.3389/fgene.2022.995163

**Published:** 2022-09-15

**Authors:** Paolo Pizzul, Erika Casari, Marco Gnugnoli, Carlo Rinaldi, Flavio Corallo, Maria Pia Longhese

**Affiliations:** Dipartimento di Biotecnologie e Bioscienze, Università degli Studi di Milano-Bicocca, Milano, Italy

**Keywords:** DNA damage, checkpoint, yeast, protein kinases, cell cycle

## Abstract

Studies performed in the yeasts *Saccharomyces cerevisiae* and *Schizosaccharomyces pombe* have led the way in defining the DNA damage checkpoint and in identifying most of the proteins involved in this regulatory network, which turned out to have structural and functional equivalents in humans. Subsequent experiments revealed that the checkpoint is an elaborate signal transduction pathway that has the ability to sense and signal the presence of damaged DNA and transduce this information to influence a multifaceted cellular response that is essential for cancer avoidance. This review focuses on the work that was done in *Saccharomyces cerevisiae* to articulate the checkpoint concept, to identify its players and the mechanisms of activation and deactivation.

## Introduction

**FIGURE 1:**
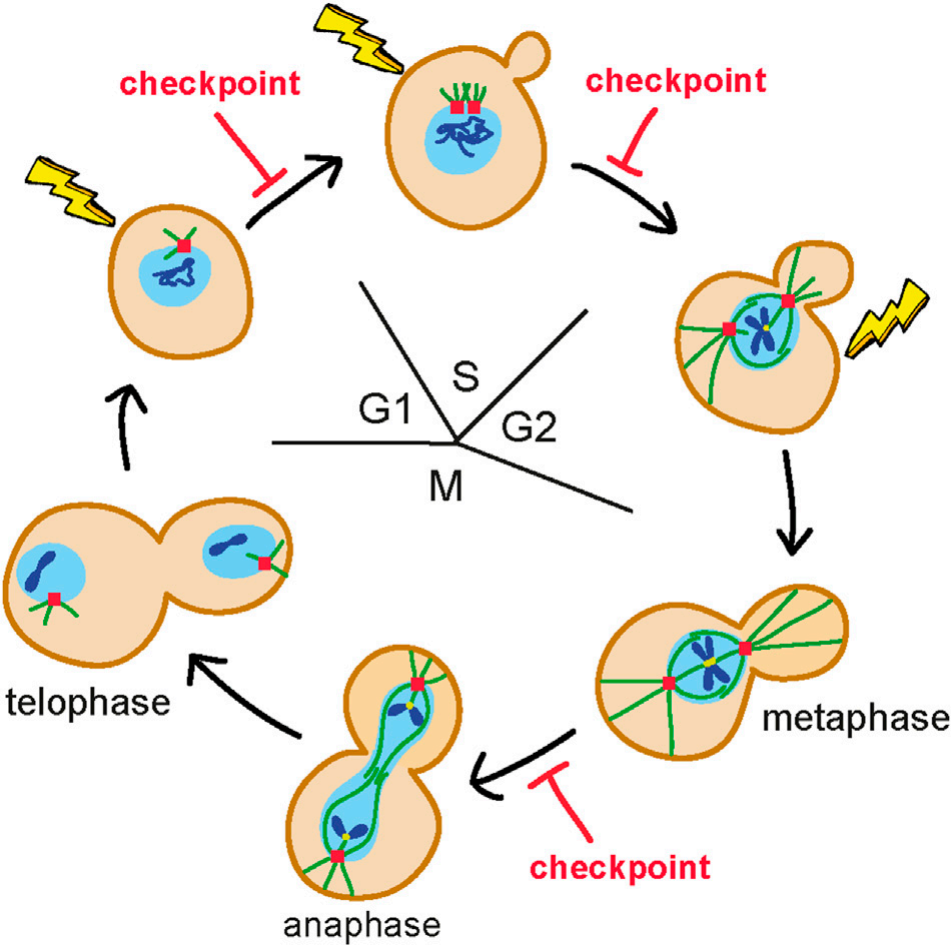
Budding yeast mitotic cell cycle. During the G1 phase, cells are unbudded and contain a single spindle pole body (SPB) (red square). Once cells become committed to enter S phase, they start budding, duplicate the SPB and replicate DNA (blue lines). In G2, the mitotic spindle (green lines) is assembled along the mother-daughter axis and the nucleus (light blue circle) moves to the bud neck. In M phase, duplicated chromosomes get attached (yellow dots) to the microtubules in a bipolar manner in metaphase and are pulled apart during anaphase. Once chromosome segregation is completed (telophase), the spindle disassembles and the 2 cells get physically separated. The nuclear envelope does not breakdown. Only one chromosome is shown. DNA damage in G1 activates a checkpoint that arrests the G1/S transition, whereas DNA damage in G2 after completion of DNA replication activates a checkpoint that arrests the metaphase to anaphase transition. Detection of DNA lesions during S phase elicits a checkpoint response that controls completion of DNA replication before cells enter M phase.

The cell cycle is a flux of events that occur at a precise time and in a defined order to allow duplication of a cell. It is divided into four phases: G1, S, G2, and M. In *Saccharomyces cerevisiae*, during the G1 phase, cells grow and become committed to enter S phase, during which they start budding, replicate DNA, and duplicate the spindle pole body (SPB), the functional equivalent of the mammalian centrosome. In G2, the mitotic spindle assembles along the mother-daughter axis and the nucleus migrates at the bud neck. Finally, during M phase, the duplicated chromosomes, attached to microtubules in a bipolar manner in metaphase, are pulled apart in anaphase (Figure 1). Once chromosome segregation is completed in telophase, the spindle disassembles and the 2 cells divide during cytokinesis.

Pioneer works in the yeasts *Saccharomyces cerevisiae* and *Schizosaccharomyces pombe* permitted to isolate temperature sensitive *cdc* (cell division cycle) mutants that affected specific cell-cycle processes, such as bud emergence, initiation of DNA synthesis, chromosome segregation, and cytokinesis (Hartwell et al., 1974; Nurse et al., 1976). Characterization of these mutants suggested that most of the cell-cycle events were ordered into dependent pathways, in which the execution of late events depended on the completion of early ones. This dependency was hypothesized to be due to either substrate-product order, in which the next event physically required the completion of the previous event, or to regulatory mechanisms that controlled the execution of one phase of the cell cycle before allowing the subsequent cell-cycle transition to occur. These regulatory mechanisms were hypothesized to belong to two classes: intrinsic mechanisms constitutively acting in each cell cycle to ensure proper temporal order of events and extrinsic mechanisms that were activated by the presence of alterations.

It had long been known that cells exposed to either UV or X-rays irradiation, or treated with genotoxic chemical compounds underwent an arrest of cell-cycle progression (Burns, 1956; Yamada and Puck, 1961; Hittelman and Rao, 1974; Tobey, 1975; Brunborg and Williamson, 1978; Busse et al., 1978). With the understanding that chemical and physical agents can change the chemical structure of DNA, a cell-cycle arrest induced by DNA lesions could be due to regulatory mechanisms that are elicited by changes in the DNA molecule. The existence of a control mechanism that “would examine cells at multiple stages within G2 to ensure that the damaged DNA was repaired normally, in which case a cell would be returned to cycle” was intuited by Tobey (1975) during the study of CHO Chinese hamster cells treated with the genotoxic drug neocarzinostatin. A DNA damage-induced control of cell-cycle progression was also hypothesized in *S. pombe* cells after UV-induced DNA damage (Hannan et al., 1976) or in irradiated human cell lines from patients suffering the ataxia-telangiectasia syndrome (Painter and Young, 1980). However, it was not until studies in the yeast *S. cerevisiae* that Weinert and Hartwell (1988) demonstrated that the arrest of the cell cycle after DNA damage was genetically controlled, and first articulated this arrest phenomenon as “checkpoint”, because it appeared to have the role of checking to ensure that prerequisites had been properly satisfied before allowing the return to cell-cycle progression (Hartwell and Weinert, 1989).

## Identification of checkpoint genes

By analyzing a collection of more than 30 *rad* (radiation-sensitive) mutants for their ability to allow cell-cycle progression after X-rays by a microcolony assay, Weinert and Hartwell (1988) found that the *RAD9* gene provoked an arrest of the G2/M transition after DNA damage, thus establishing the actively regulated nature of this arrest phenomenon and genetically defining the first checkpoint gene. Two years later, Enoch and Nurse (1990) identified the first *S. pombe* checkpoint mutant, which abolished dependence of mitosis on DNA replication. In this case, the mutation was an allele of the *CDC2* gene that encodes for the cyclin-dependent protein kinase, thus linking the checkpoint response to a key regulator of the cell cycle. Furthermore, by analyzing the ability of 20 *S. pombe rad* mutants to arrest the cell cycle in response to DNA lesions or inhibition of DNA synthesis, al-Khodairy and Carr (1992) and Enoch et al. (1992) identified the additional *RAD1*, *RAD3*, *RAD9*, *RAD17,* and *RAD24* checkpoint genes in *S. pombe*.

Another way to induce DNA alterations was to mutationally inactivate the *CDC* genes involved in DNA metabolism. In fact, some *cdc* mutants, such as *cdc9* and *cdc13* mutant cells that are defective in DNA replication and telomere protection, respectively, were shown to arrest in G2 when incubated under restrictive conditions. The finding that inactivation of the *S. cerevisiae RAD9* gene caused *cdc13* mutant cells to continue the cell cycle and to rapidly lose viability was used by Weinert et al. (1994) as a genetic strategy to identify other checkpoint genes. In fact, the search for mutations that caused rapid cell death and failure of *cdc13* cells to arrest the cell cycle after shift to the restrictive temperature, allowed the identification of the three checkpoint genes *MEC1*, *MEC2* (also known as *RAD53*) and *MEC3* (for Mitosis Entry Checkpoint).

While Mec3 was found to be involved in arresting the G2/M transition after DNA lesions, Mec1 and Mec2/Rad53 turned out to be essential also to prevent entry into mitosis in the presence of incompletely replicated DNA, suggesting an involvement of the checkpoint in supporting DNA replication under stress conditions. Consistent with a role of the checkpoint during a perturbed S phase, two other checkpoint genes, *DDC1* and *DDC2* (for DNA Damage Checkpoint), were identified as mutations causing cell death when combined with a mutant affecting the DNA primase subunit of the polα-primase complex (Longhese et al., 1997; Paciotti et al., 1998, 2000). *DDC2* was also independently identified in a search for open reading frames with homology to *S. pombe* Rad26 (Rouse and Jackson, 2000) and in a two-hybrid screen for Mec1 interactors (Wakayama et al., 2001).

While Mec2/Rad53 turned out to be a protein kinase that had been identified 3 years earlier in a biochemical screen by Stern et al. (1991), *MEC1* was also found by Kato and Ogawa (1994) by searching for mutants that were hypersensitive to methyl methanesulfonate (MMS) and defective in meiotic recombination. Furthermore, both *RAD53* and *MEC1* genes were identified as *SAD1* and *SAD3* (for S-phase Arrest-Defective), respectively, also by Elledge’s laboratory in a screen for mutants that conferred sensitivity to the DNA synthesis inhibitor hydroxyurea and whose sensitivity could be suppressed by preventing initiation of DNA replication (Allen et al., 1994). Sanchez et al. (1996) also first showed that Mec2/Rad53 was phosphorylated by Mec1 and by another kinase, Tel1, previously identified among a collection of temperature sensitive mutants with altered telomere length (Lustig and Petes, 1986).

Subsequent studies have shown that checkpoint activation can arrest different cell-cycle transitions depending on the phase of the cell cycle in which DNA lesions are detected (Figure 1). In fact, DNA damage in G1 activates a checkpoint response that arrests the G1/S transition (Siede et al., 1994), whereas detection of DNA damage during S phase elicits a checkpoint that controls completion of DNA replication before cells enter M phase (Paulovich and Hartwell, 1995). Finally, if DNA lesions are detected in G2, they activate a checkpoint that arrests the metaphase to anaphase transition (Weinert et al., 1994; Vaze et al., 2002).

## Apical checkpoint kinases

**FIGURE 2:**
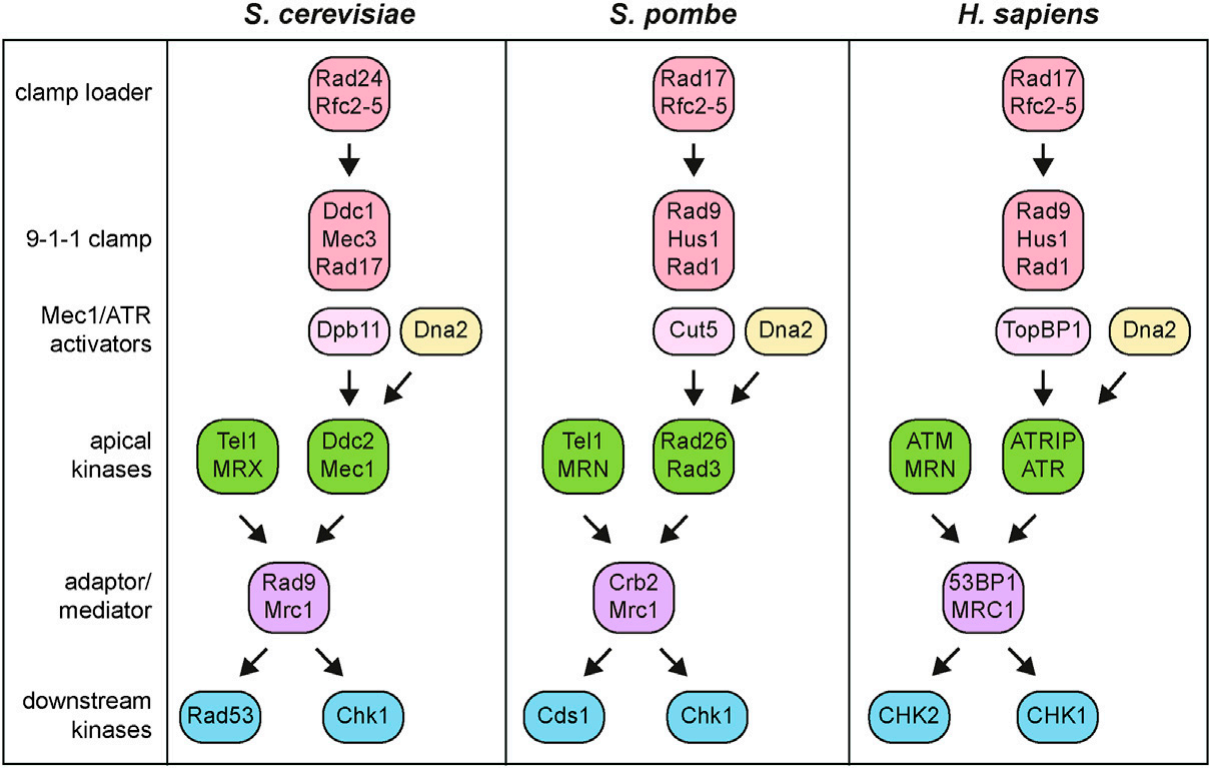
Simplified DNA damage checkpoint architecture in *S. cerevisiae*, *S. pombe* and *H. sapiens*. See text for details.

Following the definition of the checkpoint by Weinert and Hartwell in 1988 as “control mechanisms enforcing dependency in the cell cycle” (Hartwell and Weinert, 1989), subsequent genetic and biochemical studies established that the checkpoint elicited by DNA lesions was a network of highly conserved signal transduction pathways (Figure 2). These pathways not only restrain chromosome segregation until the damaged DNA has been fixed, but are integrated into a multifaceted DNA damage response. Similar to other signaling pathways, the DNA damage checkpoint is composed of sensors, which are able to recognize the presence of DNA lesions or aberrant DNA structures and transmit a signal throughout transducers to a set of effectors that participate in a broad range of cellular processes (Elledge, 1996), including inhibition of origin firing, protection and stability of replication forks, regulation of deoxyribonucleotide (dNTP) production, induction of transcription, control of DNA repair, and initiation of apoptosis or autophagy (Lanz et al., 2019).

One of the most striking features of these signal transduction cascades are the presence of apical protein kinases that in yeast are represented by Tel1 and Mec1 (Figure 2). These two proteins belong to the phosphatidylinositol-3-kinase-related kinase (PIKKs) family, whose recruitment to damaged DNA initiates checkpoint activation. In particular, Tel1 turned out to be ortholog of the human ATM (ataxia-telangiectasia mutated) gene, whose mutation is responsible for the autosomal recessive disorder ataxia-telangiectasia (Greenwell et al., 1995; Morrow et al., 1995; Savitsky et al., 1995), whereas Mec1 was the ortholog of human ATR (ATM and Rad3-related) gene, whose mutation causes the Seckel syndrome (O'Driscoll et al., 2003). A screen for *S. cerevisiae* mutations in *TEL1* gene that compensated for the lack of Mec1 function showed that Mec1 and Tel1 have partially overlapping functions (Baldo et al., 2008). These *tel1* alleles, called *tel1-hy* (hyperactive), partially bypassed Mec1 function in allowing Rad53 activation and in supporting cell survival in the presence of DNA damaging agents. While suppression of some Tel1-hy mutant variants was due to an enhanced kinase activity, changes in the ability to interact with specific protein targets or with damaged DNA could be the reason for the suppression of the Tel1-hy mutant variants that showed similar or even lower kinase activity compared to wild-type Tel1.

Once activated, Tel1/ATM and Mec1/ATR phosphorylate the downstream checkpoint kinases Rad53 (*S. pombe* Cds1 and human CHK2) and Chk1 (*S. pombe* and human CHK1), which control two parallel branches of the checkpoint by catalyzing downstream phosphorylation events (Figure 2) (Sanchez et al., 1999).

**FIGURE 3:**
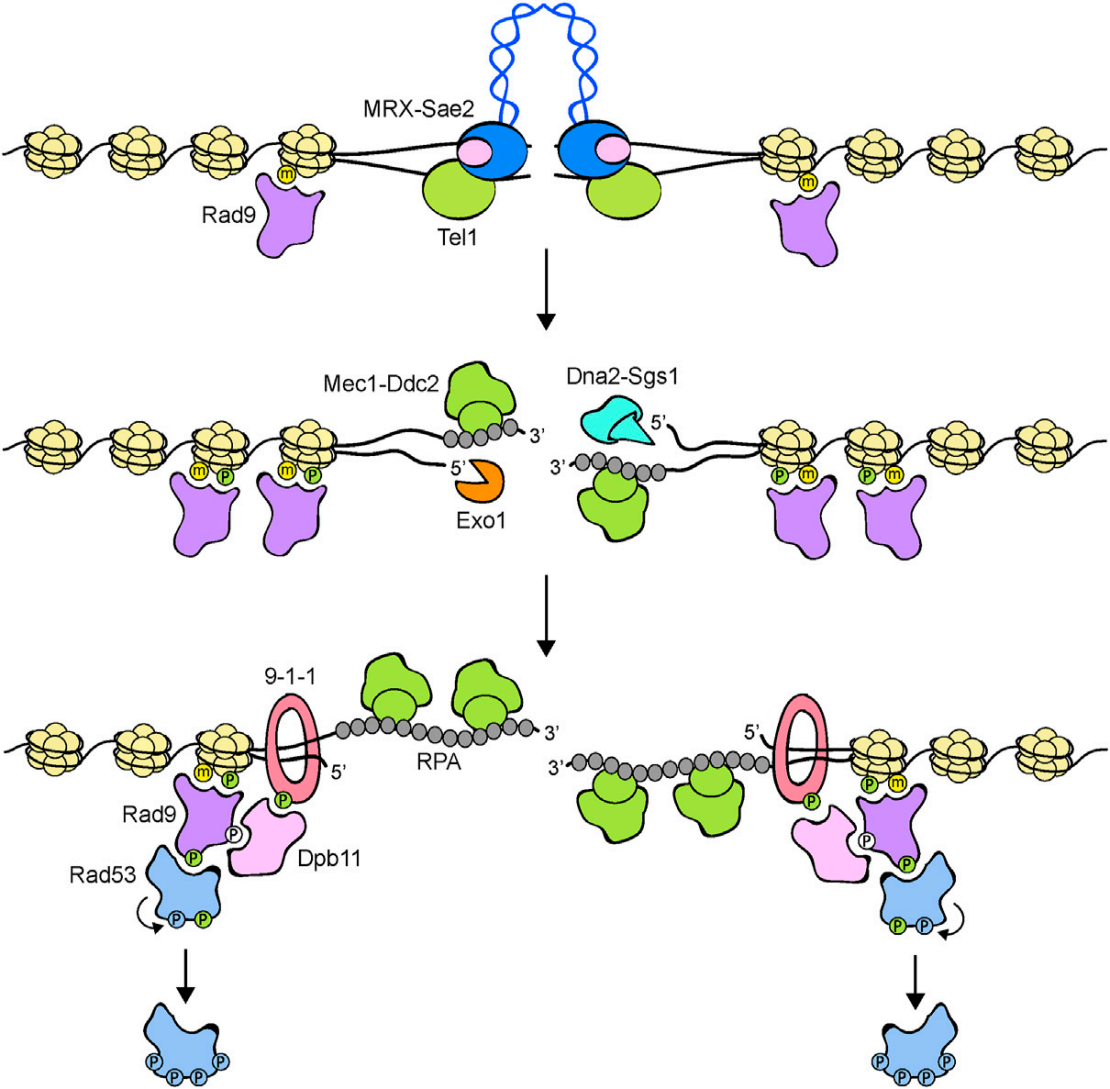
Model for Rad53 activation in response to DNA DSBs. The MRX-Sae2 complex is rapidly recruited to DNA ends. Rad9 is already bound to chromatin via interaction with methylated histone H3 (yellow dots). MRX bound to DNA ends recruits and activates Tel1, which in turn phosphorylates histone H2A on S129 (green dots), an event that leads to a further enrichment of Rad9 at DSBs. DSB end processing by Exo1 and Dna2-Sgs1 nucleases generates ssDNA that is coated by RPA. RPA-coated ssDNA allows the recruitment of Mec1-Ddc2 and a switch from Tel1 to Mec1 signaling. The 9-1-1 clamp loader recruits the 9-1-1 complex at the 5′ recessed end of the ssDNA-dsDNA junction. Mec1 in turn phosphorylates the Ddc1 subunit of the 9-1-1 complex (green dots), thus creating a docking site for Dpb11 binding. Rad9, once phosphorylated by Cdk1 (white dots), can also bind to Dpb11 that acts as a scaffold to promote Rad9-Mec1 interaction and therefore Rad9 phosphorylation by Mec1. Phosphorylated Rad9 first acts as an adaptor to bring Rad53 into close proximity to Mec1 to allow Mec1-dependent Rad53 phosphorylation. Then, Rad9 promotes Rad53 in trans-autophosphorylation (light blue dots) by increasing the local concentration of Rad53 molecules. Fully activated Rad53 molecules are then released from the Rad9 complex. Rad9 oligomerization is not shown.

**Tel1 protein kinase.** Tel1 was originally identified in the budding yeast *S. cerevisiae* for its requirement to maintain telomere length (Lustig and Petes, 1986). Subsequent work has shown that Tel1, as well as its human ortholog ATM, is the apical kinase involved in signaling unprocessed or minimally processed DNA double-strand breaks (DSBs). In both yeast and mammals, recruitment and activation of Tel1/ATM requires the MRX (human MRN) complex (Nakada et al., 2003; Uziel et al., 2003; Lee and Paull, 2004, 2005; Falck et al., 2005; You et al., 2005; Duprè et al., 2006), which is composed of the Mre11, Rad50 and Xrs2 (human MRE11, RAD50 and NBS1) subunits and plays a key role in recognition and processing of DNA ends (Syed and Tainer, 2018) (Figures 2, 3).

Mre11 has both 3′-5′ exonuclease and dsDNA endonuclease activities, whereas Rad50 is an ATPase belonging to the ABC transporter family (Paull and Gellert, 1998; Trujillo et al., 1998; Syed and Tainer, 2018). Two Mre11 nucleases and two Rad50 nucleotide binding domains interact to each other to form a globular head that possesses DNA binding and processing activities. Two long Rad50 anti-parallel coiled-coil arms protrude from the head domain to form a ring- or rod-like structure that can dimerize through a Zn-hook motif (Syed and Tainer, 2018) (Figure 3).

Rad50 ATPase activity drives conformational changes of the complex that modulate the different MRX functions. In fact, when bound to ATP, the Rad50 dimer forms a DNA binding platform that prevents Mre11 to access to dsDNA (Williams et al., 2008, 2011; Lammens et al., 2011; Möckel et al., 2012; Deshpande et al., 2014). By contrast, ATP hydrolysis induces a dissociation of the Rad50 nucleotide binding domains that allows DNA to access to the Mre11 nuclease active sites (Lammens et al., 2011). A recent high-resolution structure of the Mre11-Rad50 complex from *Escherichia coli* shows that the MRX encountering of DNA ends induces a ring-to-rod transition of the Rad50 coiled-coils that allows the reposition of Mre11 to the side of Rad50 dimer to generate a DNA cutting channel that allows Mre11 to nucleolytically process the DSB ends (Käshammer et al., 2019).

The Xrs2 subunit, which is only present in eukaryotes, interacts with Tel1/ATM and recruits it to both telomeres and DSBs (Nakada et al., 2003; You et al., 2005). However, also Mre11 and Rad50 interact individually with Tel1 in both *S. cerevisiae* and *S. pombe* and these interactions do not require Xrs2/NBS1 (Limbo et al., 2018; Hailemariam et al., 2019). Furthermore, Tel1 stimulation by each of the subunits alone and by different heterodimeric pairs showed that the Mre11-Xrs2 subcomplex fails to stimulate Tel1 kinase activity, whereas either Rad50-Mre11 or Rad50-Xrs2 subcomplex is capable to promote Tel1 activation (Hailemariam et al., 2019). These data indicate that Rad50 is essential, but not sufficient, to stimulate Tel1, whose activation requires Rad50 binding with either Mre11 or Xrs2 that possibly provides additional interactions to strengthen Rad50-Tel1 complex formation.

*In vitro* activation of human and *S. cerevisiae* Tel1/ATM requires ATP binding but not ATP hydrolysis by MRX/MRN (Lee et al., 2013; Hailemariam et al., 2019), suggesting that MRX/MRN needs to be bound to ATP to stimulate Tel1/ATM activation. The identification of *S. cerevisiae* *rad50-A78T* mutant allele, which affects Tel1 activation without impairing MRX functions in DNA damage repair, supports this hypothesis (Cassani et al., 2019). In fact, molecular dynamics simulations have shown that the ATP-bound Mre11-Rad50^A78T^ subcomplex undergoes conformational changes similar to those observed when the wild-type Mre11-Rad50 subcomplex is bound to ADP. This observation suggests that Mre11-Rad50^A78T^ fails to activate Tel1 because it is unable to maintain the conformation that is induced by ATP binding.

While MRX/MRN is required to recruit Tel1/ATM to DSBs, the presence of Tel1/ATM bound to DNA ends has a structural role in stabilizing MRX association with DNA ends. This Tel1-mediated stimulation of MRX persistence to DNA ends occurs independently of Tel1 kinase activity and is important to allow proper MRX-DNA binding that is necessary to sustain DSB repair (Cassani et al., 2016).

DNA DSBs can be repaired by homologous recombination (HR) that is initiated by nucleolytic degradation (resection) of the 5′ strand at either side of the DSB. This degradation is accomplished by a concerted action of nucleases, including Exo1 and the helicase/nuclease Sgs1-Dna2 complex (Cejka and Symington, 2021) (Figure 3). The subsequent generation of 3′-ended single-stranded DNA (ssDNA) ends not only irreversibly channels DSB repair into HR, but also inhibits Tel1 signaling activity (Mantiero et al., 2007). Similarly, mammalian ATM can be activated by blunt ends or short overhangs, whereas it is inhibited by DNA molecules possessing long ssDNA overhangs at their ends (Shiotani and Zou, 2009). Attenuation of Tel1/ATM signaling activity by ssDNA generation occurs concomitantly with activation of the Mec1/ATR kinase (Figure 3). Interestingly, the lack of Sae2 (human CtIP), a *S. cerevisiae* protein that stimulates Mre11 nuclease activity to initiate resection of the DSB ends (Cannavo and Cejka, 2014), increases MRX persistence at DSBs and enhances Tel1 activation (Usui et al., 2001; Clerici et al., 2006), suggesting that Sae2 can inhibit Tel1 activity either directly or by promoting ssDNA generation at the DSB ends.

**Mec1 protein kinase.** The Mec1 protein kinase recognizes and is activated by ssDNA tracts that are coated by the Replication Protein A (RPA) complex (Zou and Elledge, 2003) (Figure 3). These ssDNA-RPA complexes are common intermediates during DNA replication and DNA repair. Mec1 activation during S phase requires higher levels of RPA-coated ssDNA than those necessary to activate the checkpoint in G1 or in G2, suggesting the existence of a threshold for checkpoint activation during DNA replication that ensures that the ssDNA that is normally generated at functional replication forks is not enough to induce a checkpoint response (Shimada et al., 2002; Tercero et al., 2003).

The connection between the generation of ssDNA and the activation of a Mec1/ATR checkpoint has been boosted by the study of the DNA damage response elicited by the site-specific HO endonuclease. HO allows mating type switching in *S. cerevisiae* by generating a DSB in the *MAT* locus that is repaired by HR (Haber, 2016). Haber’s laboratory showed that the HO gene under the control of a galactose-inducible promoter in cells lacking the homologous donor loci *HML* and *HMR* made it possible to induce a single unrepairable DSB that is sufficient to elicit a Mec1-Ddc2-dependent checkpoint, causing an arrest of the G2/M transition (Lee et al., 1998). Furthermore, Mec1 activation parallels formation of ssDNA by 5′ to 3′ nucleolytic degradation of the DSB ends, a step that was shown to require the activity of the cyclin-dependent kinase Cdk1-Clb (Ira et al., 2004).

Mec1, as well its human ortholog ATR, exists in a stable complex as a dimer of heterodimers with Ddc2 (*S. pombe* Rad26 and human ATRIP) (Figure 2), whose lack causes the same phenotypes as loss of Mec1 (Paciotti et al., 2000; Rouse and Jackson, 2000; Wakayama et al., 2001). Ddc2 loads Mec1 to damaged DNA through its direct interaction with RPA (Rouse and Jackson, 2002; Zou and Elledge, 2003). Furthermore, it contributes to the stability of the Mec1-Ddc2 complex through Ddc2-Ddc2 dimerization (Deshpande et al., 2017) and stimulates Mec1 activation at RPA-ssDNA tracts (Biswas et al., 2019).

However, the association of Mec1/ATR-Ddc2/ATRIP with RPA-coated ssDNA is not enough to induce Mec1/ATR activation. In budding yeast, at least two pathways exist that promote Mec1 kinase activity. First, Mec1 can be activated by the multi-BRCT domain protein Dpb11 (human TOPBP1) that is recruited at the junctions between ssDNA and dsDNA by the Ddc1-Mec3-Rad17 (*S. pombe* and human Rad9-Hus1-Rad1) complex (Figures 2, 3). This complex, known as 9-1-1 clamp, is an heterotrimer with a ring-shaped structure that is recruited to DNA in a Mec1-Ddc2-independent manner by the Replication factor C (RFC)-like clamp loader Rad24-Rfc2-5 (human RAD17-RFC2-5) (Kondo et al., 2001; Melo et al., 2001; Majka et al., 2006; Navadgi-Patil and Burgers, 2009). In both yeast and mammals, the interaction between Dpb11 and 9-1-1 is induced by Mec1-mediated phosphorylation of Ddc1 (Delacroix et al., 2007; Puddu et al., 2008; Navadgi-Patil and Burgers, 2009; Pfander and Diffley, 2011). In addition to Dpb11, the Dna2 nuclease/helicase stimulates Mec1 kinase during DNA replication (Kumar and Burgers, 2013) (Figure 2). In budding yeast, Ddc1 by itself stimulates Mec1 activation, but this function is probably not present in both *S. pombe* and humans (Navadgi-Patil and Burgers, 2009).

These activators show partial redundancy in eliciting Mec1 kinase activity during the cell cycle. In fact, Ddc1 appears to mediate Mec1 activation when DNA damage occurs in G1, whereas checkpoint activation in G2 involves both Dpb11 and 9-1–1 (Navadgi-Patil and Burgers, 2009; Navadgi-Patil et al., 2011). Dna2, Dpb11, and Ddc1 all contribute to activate Mec1 during S phase (Kumar and Burgers, 2013).

The molecular details of how Ddc1, Dpb11, and Dna2 activate Mec1 are not fully understood. The artificial co-localization of Ddc2 with the checkpoint mediator Mrc1 bypasses Dpb11 and Ddc1 requirements in checkpoint activation, suggesting that, rather than promoting Mec1 catalytic activity, they can act as scaffolds to keep proteins close to each other and facilitate phosphorylation events (Berens and Toczyski, 2012).

## Downstream checkpoint kinases

Once activated, the Tel1/ATM and Mec1/ATR apical kinases phosphorylate the downstream Rad53/Cds1/CHK2 and Chk1 checkpoint kinases, which in turn phosphorylate and modulate the activity of effector proteins (Figure 2). While Mec1 activates both Rad53 and Chk1, Tel1 plays a poor role in activation of these downstream kinases (Sanchez et al., 1999). By contrast, human ATR primarily activates CHK1, while ATM activates CHK2 (Matsuoka et al., 1998). Rad53 is the principal effector kinase that mediates checkpoint activation in response to DNA damage in all the cell-cycle phases, whereas Chk1 contributes to activate only the G2/M checkpoint (Sanchez et al., 1999). Although Rad53 sequence appears to be more closely related to CHK2 (Matsuoka et al., 1998), its function is taken over by CHK1 that is the primary checkpoint effector kinase in mammals.

Signal transduction from apical to downstream checkpoint kinases requires the two mediator proteins Rad9 and Mrc1 (Figure 2). In particular, Rad9 serves as a mediator in response to DNA damage in G1 and G2, whereas Mrc1 accomplishes this role during DNA replication. Rad9, the first checkpoint protein identified in yeast by Weinert and Hartwell (1988), is recruited to chromatin by multiple mechanisms (Figure 3). In the absence of DNA damage, the Rad9 Tudor domains can recognize histone H3 methylated on lysine 79, a modification that is catalyzed by the methyltransferase Dot1 (van Leeuwen et al., 2002; Giannattasio et al., 2005; Wysocki et al., 2005; Toh et al., 2006; Grenon et al., 2007). Rad9 association with sites of damage is induced by an interaction between its BRCT domain and histone H2A (human H2AX) that has been phosphorylated at serine 129 (γH2A/γH2AX) by Mec1 and Tel1 (Downs et al., 2000; Shroff et al., 2004; Toh et al., 2006; Hammet et al., 2007). Rad9 recruitment to DNA lesions depends also on Dpb11, which acts as a scaffold that brings Rad9, 9-1-1 and Mec1-Ddc2 molecules in close proximity to facilitate Rad9 phosphorylation by Mec1 (Pfander and Diffley, 2011). Dpb11-Rad9 interaction requires Rad9 phosphorylation by the Cdk1-Clb complexes (Granata et al., 2010; Pfander and Diffley, 2011). It follows that Dpb11 cannot activate the checkpoint in the G1 phase of the cell cycle when Cdk1-Clb activity is low. Similarly, Crb2, the *S. pombe* ortholog of Rad9, interacts with Cut5/Rad4, the *S. pombe* ortholog of Dpb11, and this interaction is regulated by Cdk1 phosphorylation of Crb2 (Esashi and Yanagida, 1999; Mochida et al., 2004; Du et al., 2006).

Mec1 and Tel1 phosphorylate Rad9 in response to DNA damage on multiple sites and these phosphorylation events promote Rad9 multimerization through its BRCT domains and generate docking sites on Rad9 for Rad9-Rad53 interaction (Figure 3) (Sun et al., 1996, 1998; Emili, 1998; Vialard et al., 1998; Durocher et al., 1999; Schwartz et al., 2002). The observation that expression of Rad53 to high levels in *E. coli* cells causes its autophosphorylation and activation in the absence of DNA damage leads Gilbert et al. (2001) to hypothesize that increased local concentration of Rad53 was enough for its activation. Furthermore, the analysis of hydrodynamic Rad9 complexes isolated from UV-damaged *S. cerevisiae* cells showed that Rad9 phosphorylation by Mec1 facilitates Rad53 *in trans*-autophosphorylation and the release of activated Rad53 from Rad9 (Gilbert et al., 2001). These observations led to the so-called solid-state catalyst model, whereby phosphorylated Rad9 acts as a scaffold that brings Rad53 molecules close to each other to facilitate Rad53 *in trans*-autophosphorylation and subsequent release from Rad9 of activated Rad53.

An implication of this model was that Mec1 and Tel1 would be only required for providing docking sites on Rad9 for Rad53 binding and might not necessarily activate directly Rad53. However, this model did not explain the finding that Rad53 is phosphorylated by Mec1 independently of Rad53 kinase activity (Sun et al., 1996; Pellicioli et al., 1999; Lee et al., 2003) and that a Rad53-Ddc2 fusion protein can partially bypass the requirement for Rad9 in Rad53 activation (Lee et al., 2004). Subsequent biochemical reconstitution experiments made by Sweeney et al. (2005) demonstrated that Mec1 directly phosphorylates Rad53 even if Rad53 kinase activity is inactive. Furthermore, a mutation analysis demonstrated that direct phosphorylation of Rad53 by Mec1 was necessary to activate Rad53 as a protein kinase. These data lead to the adaptor-based model, whereby Mec1-dependent Rad9 phosphorylation triggers Rad53-Mec1 interaction and therefore Rad53 phosphorylation by Mec1 (Sweeney et al., 2005).

Considering that Mec1 and Tel1 phosphorylate Rad53 and Rad9 at multiple sites (Smolka et al., 2005; Sweeney et al., 2005), the above models could be combined in an adaptor-catalyst stepwise process in which Rad9, once phosphorylated by Mec1 or Tel1, acts first as adaptor to induce Mec1-Rad53 interaction and Mec1-mediated Rad53 phosphorylation at the sites of DNA lesions. Then, Rad9 acts as a scaffold to bring Rad53 molecules in close proximity to allow Rad53 *in trans*-autophosphorylation. Fully activated Rad53 molecules are then released from phosphorylated Rad9 (Figure 3).

In addition to mediate the interaction between Rad9 and Rad53, phosphorylation by Mec1 or Tel1 of the Rad9 cluster domain (SCD) induces Rad9 oligomerization at sites of damage by promoting an interaction between the BRCT and phosphorylated SCD domains (Usui et al., 2009). Impairment of Rad9 oligomerization allows Rad53 activation but impairs checkpoint maintenance, indicating that oligomerization is required to support checkpoint signaling (Usui et al., 2009). Once activated, Rad53 attenuates BRCT-SCD-mediated interaction by phosphorylating the Rad9 BRCT domain, thus promoting Rad9 dissociation from sites of damage and the turning off of the checkpoint in a negative feedback loop (Usui et al., 2009).

Whereas Rad9 allows checkpoint activation in response to DNA damage in the G1 and G2 phases, Mrc1, which is a component of the replisome, promotes Rad53 activation during DNA synthesis (Alcasabas et al., 2001; Tanaka and Russell, 2001; Katou et al., 2003; Osborn and Elledge, 2003). Reconstitution of Rad53 activation using purified Mec1 and Mrc1 showed that, instead of increasing Mec1 catalytic activity, Mrc1 facilitates phosphorylation of Rad53 by Mec1 by positively influencing their enzyme-substrate interaction (Chen and Zhou, 2009).

## Checkpoint recovery and adaptation

Once DNA lesions are repaired, cells turn off the checkpoint and resume cell-cycle progression in a process known as recovery. Recovery is accompanied by the appearance of unphosphorylated Rad53 (Vaze et al., 2002). Loss of Rad53 phosphorylation does not require protein synthesis (Tercero et al., 2003), suggesting that activated Rad53 is dephosphorylated, not degraded. Consistent with this hypothesis, a number of phosphatases are involved in checkpoint deactivation in response to different genotoxic agents, possibly by removing activatory phosphorylation events from key checkpoint components. In particular, the PP1 phosphatase Glc7 promotes Rad53 deactivation after treatment with hydroxyurea (Bazzi et al., 2010), while the PP2C phosphatases Ptc2 and Ptc3, and the PP4 phosphatase Pph3 have been shown to dephosphorylate Rad53 after generation of a persistent DSB (Leroy et al., 2003; Keogh et al., 2006; Guillemain et al., 2007). Interestingly, Pph3, in complex with Psy2 and Psy4, promotes dephosphorylation of γ-H2A (Keogh et al., 2006), whereas in complex only with Psy2 binds and dephosphorylates Rad53 upon exposure to MMS (O’Neill et al., 2007; Szyjka et al., 2008). The Pph3-Psy2 complex also interacts with the Mec1-Ddc2 complex and promotes dephosphorylation of many Mec1 targets including Mec1 itself (Hustedt et al., 2015). Pph3, Ptc2, and Ptc3 possess partial redundant functions. In fact, *pph3 ptc2 ptc3* triple mutant cells are unable to deactivate Rad53 kinase after MMS treatment, suggesting that Ptc2 and Ptc3 can substitute Pph3 function in Rad53 dephosphorylation (Travesa et al., 2008). Furthermore, cells lacking all the three phosphatases exhibit synergistic sensitivities to DNA damaging agents and defective DNA damage repair, whereas single or double deletion does not (Kim et al., 2011).

While phosphatases can deactivate already activated Rad53, cells use the scaffold protein complex Slx4-Rtt107 to counteract *de novo* Rad53 activation through a phosphatase-independent mechanism. The *SLX4* gene was originally identified in budding yeast for its requirement to support cell viability in the absence of the helicase Sgs1 (Mullen et al., 2001). Cells lacking Slx4 show persistent Rad53 phosphorylation after MMS treatment or induction of a single unrepaired DSB, suggesting a role for Slx4 in terminating Rad53 signaling (Ohouo et al., 2013; Dibitetto et al., 2016). Upon DNA lesions, Slx4 interacts with the multi-BRCT domain protein Rtt107 to form a protein complex that competes with Rad9 for interaction with Dpb11 and γ-H2A (Ohouo et al., 2013), suggesting that Slx4 downregulates Rad53 signaling by counteracting Rad9 engagement at DNA lesions.

Conceptually distinct from the process of recovery whereby cells reenter the cell cycle after having repaired the damaged DNA, cells can turn off the checkpoint response even if the DNA damage is still present. This process, known as adaptation, has been first identified in *S. cerevisiae* by Sandell and Zakian (1993), who placed the recognition site of the HO endonuclease immediately adjacent to the dispensable left telomere of chromosome VII, such that the telomere can be eliminated by HO expression. Elimination of the telomeric DNA upon HO induction caused a checkpoint-mediated cell-cycle arrest, but many cells were capable to continue cell-cycle progression without having repaired the damaged chromosome.

In budding yeast, a single unrepairable DSB is sufficient to induce a strong checkpoint activation that persist approximately a dozen hours, after which cells decrease Rad53 phosphorylation and resume cell division (Lee et al., 1998; Pellicioli et al., 2001). While cells can adapt to a single HO-induced DSB, the generation of two DSBs prevent adaptation (Lee et al., 1998), suggesting that the ability of cells to adapt depends on the strength of the checkpoint response.

A search for yeast mutants that were unable to adapt to a DNA damage-induced cell-cycle arrest showed that adaptation is prevented by a mutation in the polo kinase Cdc5, in the regulatory subunits Ckb1 and Ckb2 of casein kinase II (CKII) (Toczyski et al., 1997), and in the recombination proteins Tid1 (Rdh54) and Srs2 (Lee et al., 2001; Vaze et al., 2002). In particular, Ckb1 and Ckb2 were shown to mediate the interaction between Rad53 and Ptc2 (Guillemain et al., 2007), suggesting that loss of Ckb proteins prevents adaptation by lowering Rad53-Ptc2 interaction and therefore Rad53 dephosphorylation. The FHA (forkhead-associated) domain of Ptc2 can be phosphorylated by CKII and mutation of this CKII phosphorylation site leads to impairment of Rad53 dephosphorylation (Guillemain et al., 2007), suggesting that Ptc2 needs to be phosphorylated by CKII to dephosphorylate Rad53.

How the checkpoint is extinguished during adaptation is not fully understood. Impairment of DSB resection by elimination of Sae2, Sgs1, or Dna2, or of both Sgs1 and Exo1 impairs adaptation (Clerici et al., 2006; Eapen et al., 2012), raising the possibility that resection of DNA ends contributes to extinguish the checkpoint signal. Interestingly, the adaptation defect of *sae2*Δ, *sgs1*Δ and *exo1*Δ *sgs1*Δ mutants is due to persistent Tel1 signaling activity (Eapen et al., 2012; Clerici et al., 2014), whose unscheduled activation appears to be due to increased MRX association with the DSB ends. Consistent with this hypothesis, a persistent Tel1-dependent checkpoint can be induced by the lack of Ku70-Ku80 complex, which increases the amount of MRX bound to DSBs without impairing DSB resection (Clerici et al., 2008; Clerici et al., 2014). These observations lead to the hypothesis that a DSB resection defect can lead to a persistent Tel1 signaling activity because it favors the generation of DNA structures that are recognized by MRX. The finding that this checkpoint persistence is due to Tel1 and not to Mec1 activity suggests that cells can deactivate Mec1 more easily than Tel1. In this scenario, the function of DSB resection in turning off MRX/Tel1 signaling activity is important to allow a proper termination of the checkpoint response.

It was recently shown that Mec1 autophosphorylation at serine 1964 is necessary to turn off the checkpoint (Memisoglu et al., 2019). This modification, which is detected several hours after Mec1 is activated, has been proposed to induce a delocalization of the Mec1-Ddc2 complex from the sites of DNA lesions. Moreover, Ddc2, which is a stable protein, is degraded when cells adapt and its degradation involves phosphorylation of two Ddc2 serine residues (Memisoglu et al., 2019). Thus, inhibition of the Mec1-Ddc2 kinase by Mec1 autophosphorylation and decrease of Ddc2 abundance plays a role in adaptation.

## Checkpoint and cancer

It had long been known that DNA lesions cause mutations that can lead to carcinogenesis (Negrini et al., 2010; Jeggo et al., 2016). Several hereditary cancer predispositions result from mutations in genes involved in DNA damage repair. Furthermore, malignant cells frequently acquire loss-of-function mutations in DNA repair genes which favor disease progression and/or therapy resistance. Although DNA repair defects are clearly causative of cancer, the increased DNA damage sensitivity of cancer cells has been exploited therapeutically through the use of radio- and chemo-therapies that force their DNA damage-induced death.

What emerged more slowly was the understanding that the DNA damage checkpoint, as distinct from repair pathways *per se*, provides a barrier that cells must overcome to become malignant. In fact, the checkpoint response is often activated in preneoplastic cells in response to oncogene-induced replication stress and its loss allows these cells to proliferate with increased genome instability that entails them to become cancerous (Bartkova et al., 2005, 2006; Gorgoulis et al., 2005; Di Micco et al., 2006). Consistently, ATM is frequently mutated in a variety of cancer types (Weber and Ryan, 2015), whereas mutations in ATR promote development of melanoma and oropharyngeal cancer syndrome (Tanaka et al., 2012; Chen et al., 2017). Furthermore, inherited mutations in the human checkpoint gene TP53 predispose individuals to cancer, whereas its somatic mutations are the most frequently occurring in all tumor types (Olivier et al., 2010).

Although the high frequency of checkpoint inactivation in tumors indicates that this pathway acts as tumor suppressor, the inhibition of cell-cycle progression induced by checkpoint activation reduces the efficacy of DNA damage-induced therapies, raising the possibility that checkpoint inhibition can sensitize cancer cells to some anticancer therapies. For this reason, several inhibitors of the checkpoint kinases ATR, ATM, CHK1 and CHK2 have been developed to be used as chemo- or radio-sensitizers and some of them are currently undergoing clinical trial testing either as monotherapy or in combination with DNA damaging agents (Weber and Ryan, 2015; Pilié et al., 2019; Choi and Lee, 2022).

Inhibitors of checkpoint kinases can be used also to induce death or to increase the DNA damage sensitivity of cancer cells with DNA repair defects. This approach of synthetic lethal/synthetic cytotoxic interactions, first proposed by Hartwell et al. (1997), is based on the hypothesis that cancer cells with DNA repair defects become dependent on a compensatory mechanism for survival. Thus, inhibition of this “backup” pathway can kill them more selectively and efficiently than conventional DNA damage-induced therapies (Pearl et al., 2015). The demonstration that synthetic lethal/synthetic cytotoxic interactions can be a suitable approach was first shown in tumors with defective HR, such as those caused by germline mutations in BRCA1 and BRCA2 genes. BRCA1- or BRCA2- deficient tumor cells were shown to be highly sensitive to pharmaceutical inhibition of poly (ADP-ribose) polymerase 1 (PARP1), a protein involved in the repair of single-strand DNA breaks (SSBs) (Bryant et al., 2005; Farmer et al., 2005). The current model posits that PARP inhibition leads to the accumulation of SSBs, which are converted into DSBs upon encountering of DNA replication forks. Inhibition of PARP, in combination with HR defects due to BRCA1/BRCA2 dysfunction, results in accumulation of persistent DSBs that leads to genomic instability, mitotic catastrophe and cell death. In addition to PARP, oncogene-induced replication stress activates the ATR-CHK1 checkpoint pathway, raising the possibility to exploit the use of CHK1 or ATR inhibition in cancers harboring activated oncogenes (Rundle et al., 2017).

## Conclusion

Since the first observations that DNA damage elicited an arrest of cell-cycle progression and the checkpoint concept was proposed, our understanding of this regulatory cascade has greatly increased. It is now clear that the control of cell-cycle progression is only one of the goals orchestrated by this mechanism, which is a highly conserved network of hierarchically ordered proteins capable of detecting DNA lesions and transducing this information to control several DNA transactions, including DNA repair capacity, inhibition of origin firing, protection and stability of replication forks, control of dNTP production, and commitment to apoptosis or senescence. Furthermore, accumulating knowledge indicate an important role for the apical and downstream checkpoint kinases in orchestrating DNA repair through phosphorylation of several substrates (Lanz et al., 2019).

It is now clear that tumor progression necessitates the downregulation of the DNA damage checkpoint to achieve uncontrolled proliferation and the adaptability associated with aggressive tumors. Tumor genome profiling by deep sequencing has demonstrated that genes involved in the checkpoint response are frequently mutated in all cancer types. Furthermore, upregulation of the DNA damage checkpoint occurs in precancerous lesions, suggesting that mutations lead to oncogene activation, which in turn causes replication stresses that elicit a checkpoint response (Bartkova et al., 2005; Halazonetis et al., 2008). This condition, coupled with the subsequent downregulation of the checkpoint response possibly by genetic alterations, allows proliferation of damaged cells and continued genome instability, a prerequisite for cancer cells to rapidly adapt to its changing microenvironment. Although the DNA damage checkpoint players act as tumor suppressors, their inhibition can sensitize tumors to clastogenic therapies or can cause lethality to cancer cells that have DNA repair defects without harming normal cells. This approach of synthetic lethality needs a more and more mechanistic understanding of regulation and interactions of the proteins involved in the DNA damage checkpoint and of their functions in DNA repair to unmask new vulnerabilities for targeted therapeutics.
